# Differentially Expressed Genes between Carrot Petaloid Cytoplasmic Male Sterile and Maintainer during Floral Development

**DOI:** 10.1038/s41598-019-53717-x

**Published:** 2019-11-22

**Authors:** Bo Liu, Chenggang Ou, Shumin Chen, Qiongwen Cao, Zhiwei Zhao, Zengjian Miao, Xiaoping Kong, Feiyun Zhuang

**Affiliations:** 1 Key Laboratory of Horticultural Crop Biology and Germplasm Innovation, Ministry of Agriculture; Institute of Vegetables and Flowers, Chinese Academy of Agricultural Science, No. 12 Nanda Street, Zhongguan Cun, Haidian District Beijing, 100081 China; 2 Xining Institute of Vegetables, Xining, No. 4 Weisan Road, Biological Industry Park, Xining, 810016 Qinghai China

**Keywords:** Plant molecular biology, Plant genetics

## Abstract

Petaloid cytoplasmic male sterility (CMS) is a maternally inherited loss of male fertility due to the complete conversion of stamens into petal-like organs, and CMS lines have been widely utilized in carrot breeding. Petaloid CMS is an ideal model not only for studying the mitochondrial–nuclear interaction but also for discovering genes that are essential for floral organ development. To investigate the comprehensive mechanism of CMS and homeotic organ alternation during carrot flower development, we conducted transcriptome analysis between the petaloid CMS line (P2S) and its maintainer line (P2M) at four flower developmental stages (T1–T4). A total of 2838 genes were found to be differentially expressed, among which 1495 genes were significantly downregulated and 1343 genes were significantly upregulated in the CMS line. Functional analysis showed that most of the differentially expressed genes (DEGs) were involved in protein processing in the endoplasmic reticulum, plant hormone signal transduction, and biosynthesis. A total of 16 MADS-box genes were grouped into class A, B, C, and E, but not class D, genes. Several key genes associated with oxidative phosphorylation showed continuously low expression from stage T2 in P2S, and the expression of *DcPI* and *DcAG-*like genes also greatly decreased at stage T2 in P2S. This indicated that energy deficiency might inhibit the expression of B- and C-class MADS-box genes resulting in the conversion of stamens into petals. Stamen petaloidy may act as an intrinsic stress, upregulating the expression of heat shock protein (HSP) genes and MADS-box genes at stages T3 and T4 in P2S, which results in some fertile revertants. This study will provide a better understanding of carrot petaloid CMS and floral development as a basis for further research.

## Introduction

The utilization of cytoplasmic male sterility (CMS) lines as female parents to avoid self-pollination promotes the application of heterosis in many crops and plays a critical role in the commercial production of hybrid seeds. CMS is usually associated with unique chimeric genes resulting from mitochondrial genome rearrangements^1,2^. For instance, a chimeric mitochondrial gene named *orf224*, which is located upstream of and co-transcribed with *atp6*, was identified to be responsible for rapeseed *pol*-CMS^3^. In *Brassica juncea*, *hau*-CMS is known to contain a CMS-associated gene, *orf288*, which is located downstream of and co-transcribed with *atp6*^4^. The mitochondrial gene *orf79* has been shown to act as a CMS-associated gene in rice BT-CMS and LD-CMS, and is co-transcribed with *atp6*^5,6^. Rice WA-CMS has been shown to be associated with a novel chimerical gene, *WA352*, which inhibited the function of the nuclear-encoded mitochondrial protein COX11 and caused premature tapetal programmed cell death (PCD) and consequent male sterility^7^. It has been elucidated that these genes are responsible for CMS through a series of mechanisms including mitochondrial energy efficiency, CMS protein cytotoxicity, and premature tapetal PCD^7–9^. In the fertility line, the mitochondrial genes involved in CMS can be suppressed by nuclear *Rf* genes, most of which encode pentatricopeptide repeat (PPR) protein, leading to fertility restoration^2,10^.

In carrot (*Daucus carota* L.), two morphologically different CMS systems have been found: brown anther type and petaloid type^11,12^. Brown anther CMS, which produces shriveled and yellow-to-brown anthers, is similar to rice CMS, but is not widely utilized due to its instability^7^. However, lines with petaloid CMS, a maternally inherited loss of male fertility based on flower male organ dysfunctions due to the complete conversion of stamens into petal-like organs, have been widely utilized^12–14^. Some previous studies have been dedicated to characterizing the mechanism of carrot CMS in various ways. For example, Nakajima et.al found that rearrangement of the mitochondrial genome occurred in carrot CMS lines^15,16^. Furthermore, an *orfB*-related gene of carrot mitochondrial genomes was found to be closely associated with petaloid CMS^17^. However, later research suggested that *atp8* was most likely misidentified as *orfB* in the aforementioned study, and indicated that the expression of *atp8* was not directly associated with petaloid CMS^18^. It was reported that the ORF of *atp9* in the petaloid accession is 13 amino acids longer than that of *atp9* in the corresponding normal accession due to a point mutation, and the expression of *atp9* corresponding to the petaloid accession is higher^19^. Recently, the phenomenon of mitochondrial and nuclear DNA insertion into the plastid genome was reported in carrot^20^. CMS is known to be mainly caused by chimeras of mitochondrial *orf* genes^1^, however the mechanism of carrot petaloid CMS is still unclear.

Petaloid CMS is a relatively rare type of CMS in plants, however, stamen degeneration is not unique to carrot. Similar homeotic organ alternations have also been observed in a few other species during the progress of interspecific hybridization, somatic hybridization, and recurrent backcrossing^20,21^. For instance, the stamens of the *hau* CMS line are replaced by thickened petal-like structures in *B*. *juncea*, and *orf288* has been identified as a CMS-associated gene with higher expression level in the *hau* CMS line^9,22,23^. A novel CMS of *B*. *napus* was selected from a somatic hybrid with *Isatis indigotica* (Chinese woad) by recurrent backcrossing, resulting in the stamens being converted into carpelloid structures^24^. Flower organ alternations have also been found in interspecific cybrids between *Nicotiana tabacum* and *Hyoscyamus niger* through protoplast fusion and conventional backcrossing^25^. Interestingly, a sterile line with petaloid stamens was found in the progeny of the stem grafts of two species, *N*. *sylvestris* and *N*. *tabacum*, which carries the *N*. *undulata* cytoplasmic genome. Mitochondrial analyses showed that *orf293* was adjacent to *atp1* after interspecies mitochondrial fusion, which caused the conversion of stamens into petals^26^. Many studies have proposed that the recombinant mitochondrial gene can affect the homeotic function of nuclear genes which, in turn, alters floral development, particularly stamen loss^1,21,27,28^.

It is well established that homeotic conversion between two neighboring flower whorls is controlled by nuclear-encoded MADS-box genes^29–31^. The ABC(DE) model proposes that MADS-box genes can be grouped into five classes, A–E. Classes A, B, and E specify petal formation, while the combination of classes B, C, and E determine stamen development^32^. In ideal class B mutants, petals are substituted with sepals, and stamens are replaced by carpels. Class C mutants affect the third whorls and cause petals to develop instead of stamens^33,34^. Many studies have also demonstrated the critical role of MADS-box genes in regulating petal and stamen development in different species. *CvPI* is essential for the specification of petal and stamen identity in *Calluna vulgaris*, and its reduced gene expression has been shown to result in the transition of petals into sepals^35^. In a homeotic mutant of *Petrocosmea* (Gesneriaceae), the dosage imbalance of B- and C-class genes—due to the upregulation of the two B-class genes *DEFICIENS2* (*DEF2*) and *GLOBOSA* (*GLO*) and the downregulation of the C-class gene *PLE*—caused the formation of petaloid stamens^36^. In rose (*Rosa hybrida*), suppression of the expression of *RhAG* increased the number of petals through the conversion of stamen petaloidy^37^. Otani *et al*. (2016) developed a series of transgenic plants of *Tricyrtis* sp. with suppressed B-class genes, in which the petaloid tepals were replaced by sepaloid tepals and the stamens were converted into carpeloid structures^38^. Moreover, Dodsworth (2017) proposed a modified ABCE model, and elucidated that the formation of petaloid organs in two whorls was due to the expansion of B-class gene expression into whorl 1 in petaloid monocots^39^. Additionally, a previous study in a “carpeloid” carrot suggested that the reduced expression of *DcMADS2* and *DcMADS3*, homologs of the *Antirrhinum* genes *GLO* and *DEF*, induced the generation of homeotic floral organs^40^.

With the development of next-generation sequencing, RNA-seq was performed to identify the expression patterns of genes between CMS and normal fertile type in many plants. In *B*. *napus*, many differentially expressed genes (DEGs) participate in oxidative phosphorylation, PPR protein and anther development have been identified to be downregulated in CMS line^3^. Similarly, many DEGs involved in oxidative phosphorylation, including ATPase, NADH dehydrogenase, and cytochrome *c* oxidase, have been found to be associated with CMS through comparative analysis between onion CMS line and its maintainer^41^. Mei *et al*. (2016) indicated that some DEGs encoding MYB and bHLH transcription factors, PPR proteins, and heat shock proteins could be associated with radish CMS^42^. Ye *et al*. (2017) suggested that phenylpropanoid biosynthesis and jasmonate biosynthesis pathways play essential roles in the fertility conversion of wheat CMS^43^. Comparative transcriptomic analysis identified key genes involved in stamen petaloidy in lotus (*Nelumbo nucifera*); most of these genes were associated with hormonal signal transduction pathways and transcription factors, and the MADS-box genes were also identified as candidate genes^44^. These studies provided a good foundation for better understanding and studying the regulatory network of CMS and flower organ development. Recently, a high-quality genome sequence of carrot was assembled and published, which is convenient for studying the mechanisms of CMS at a genome level^45^. In this study, we conducted transcriptomic analysis of carrot umbels from the petaloid CMS line and its corresponding maintainer line at four development stages. By comparing the variation of transcript profiles between the petaloid CMS line and its maintainer line, some candidate genes associated with flower development and CMS were identified and discussed. This study will provide an improved understanding of the regulatory network in flower development and CMS in carrot.

## Results

### Phenotypic analysis of floral development in the petaloid cytoplasmic male sterility (CMS) line (P2S) and its maintainer line (P2M)

**Figure 1:**
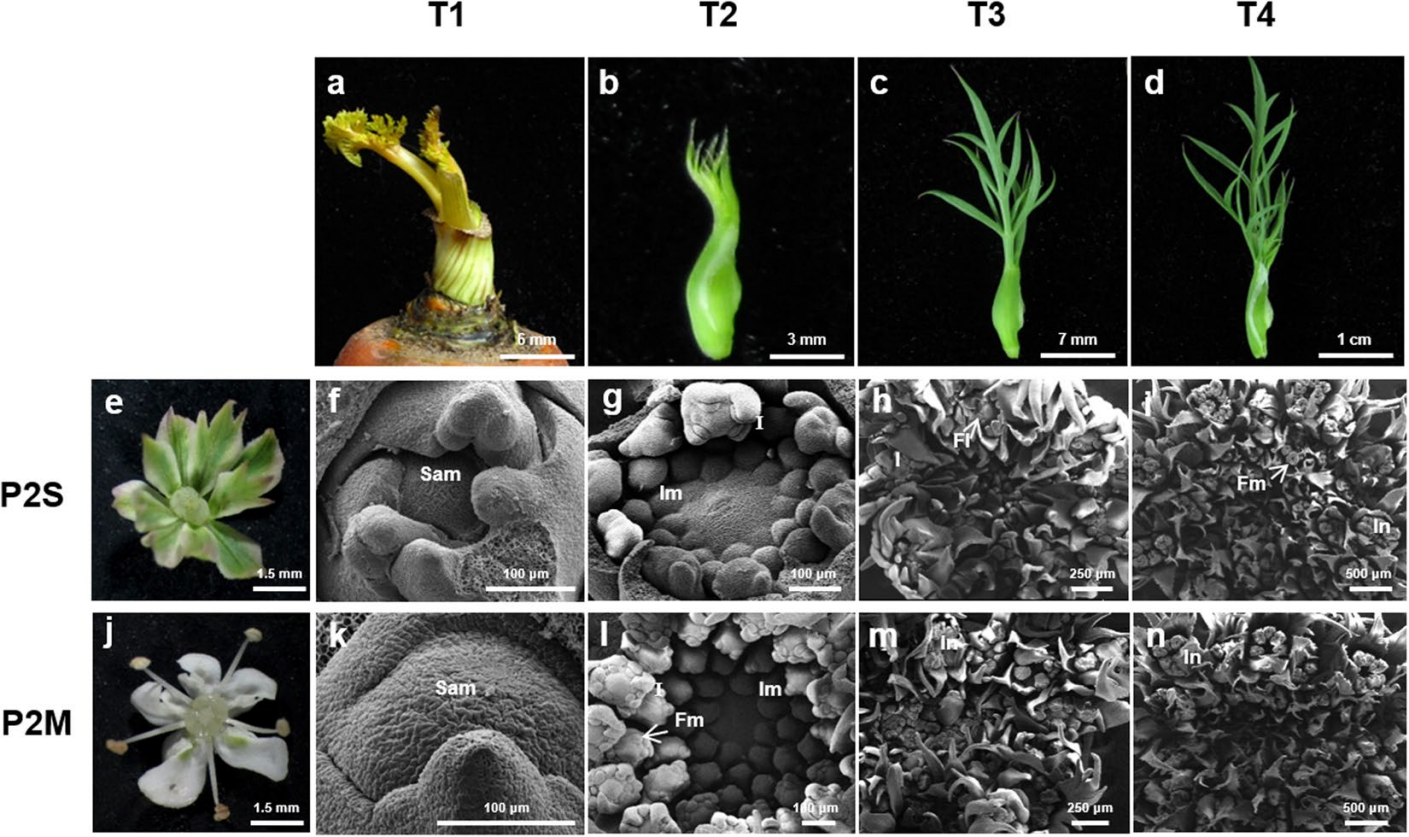
Morphological observation of floral development at four stages of P2S and P2M. (**a**,**f**,**k**) Shoot apical meristem germination at T1; (**b**,**g**,**l**) Inflorescence about 2 mm in diameter at T2; (**c**,**h**,**m**) Inflorescence about 3 mm in diameter at T3; (**d**,**i**,**n**) Inflorescence about 4 mm in diameter at T4. (**e**) The flower of CMS P2S and (**j**) that of maintainer line P2M. **Sam**, Shoot apical meristem; **Im**, Inflorescence primordium; **In**, Inflorescence; **Fm**, Floret primoridium; **FL**, floret.

To enable more detailed detection of the differentiation process in floral development, we compared the morphological change of umbels from P2S (Fig. 1e–h) and P2M (Fig. 1j–m) at four developmental stages by scanning electron microscopy. The size of floral buds was sampled according to the previous phenotype observation^40^. At stage T1, only one shoot apical meristem germinated in the middle of the stem apex (Fig. 1e,j). During stage T2, some young inflorescences had appeared at the periphery of the umbel with multiple germinated inflorescence primordia in the center (Fig. 1f,k). At stage T3 which was similar with the stages s2-s3^40^, the inflorescence gradually became mature and many floret primordia germinated in the middle of the inflorescence (Fig. 1g,l). At stage T4, floret primordia had developed into young florets in each inflorescence and formed whole compound umbels (Fig. 1h,m). There was no obvious morphological difference between P2S and P2M in the four stages. However, when the flowers opened, the stamens of P2M normal fertile flowers were substituted by petals in P2S (Fig. 1n,i). Additionally, the real petals of P2S were a greenish color, different to that of the normal white petals of P2M. It is interesting that about 3.4% (15/440) of revertant plants with normal stamens were found in P2S.

### Sequence assembly and functional annotation of differentially expressed genes (DEGs)

The gene regulation of stamen and petals development is initiated earlier than the organs morphological differentiation^31^. In order to explore the regulatory networks related to carrot CMS, we performed RNA-seq analysis of umbels from P2S and P2M at four stages. After Illumina sequencing and data processing, a total of 118.03 Gbp of clean data was obtained, with the data of each sample occupying more than 4.13 Gbp. The Q30 base percentage of all samples was above 88.65% and the average GC content was 44.92%, indicating that the quality of sequencing data was sufficient for the subsequent analysis of DEGs. Then, these clean reads were mapped to the carrot reference genome, and fragments per kilobase of transcript per million fragments mapped (FPKM) was used to evaluate the expression level of each gene. An average of 24,678; 24,438; 24,539; and 24,529 P2S genes were identified as expressed at stages T1, T2, T3, and T4, respectively, and an average of 25,212; 23,942; 25,571; and 24,768 P2M genes were identified as expressed at stages T1, T2, T3, and T4, respectively. Additionally, 1319 novel transcripts were detected in this work, 861 of which were functionally annotated in public databases (Table S1).

**Figure 2:**
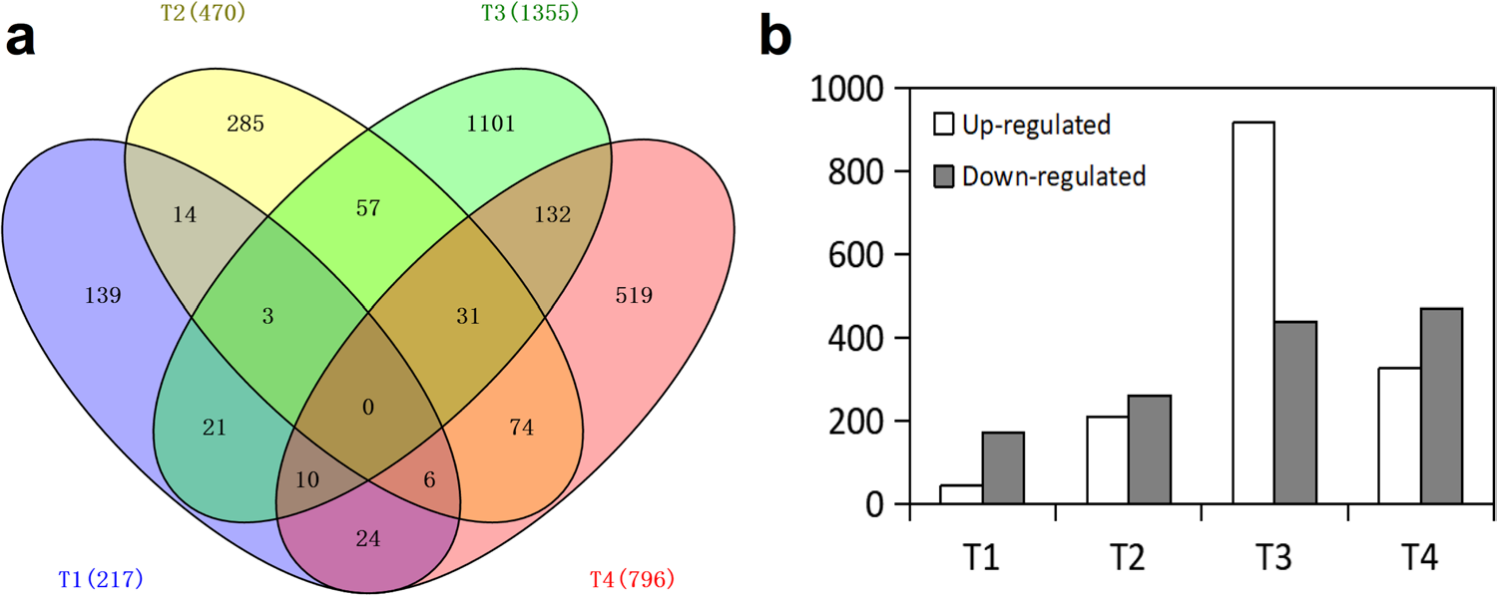
Overview of differentially expressed genes (DEGs) between P2M and P2S. (**a**) Venn diagram of DEGs showing the overlaps between P2S and P2M at four stages. (**b**) Histogram showing the number of DEGs between P2S and P2M at four stages.

A false discovery rate (FDR) of <0.01 and fold change of >2 were used as the thresholds for screening DEGs between P2S and P2M libraries. The analysis identified a total of 2838 genes as being differentially expressed at the four flower developmental stages, of which 1495 were significantly upregulated and 1343 were significantly downregulated in P2M compared to P2S (Fig. 2). Compared to P2S, 217, 470, 1355, and 796 P2M genes were identified as being differentially expressed at stages T1, T2, T3, and T4, respectively (Fig. 2a). Additionally, 31 genes were found to be significantly differentially expressed between P2S and P2M at the latter three stages, 11 of which were heat shock proteins (Table S2). Three genes were differentially expressed in the previous three stages, but no genes for all of the four stages (Fig. 2a). Compared with P2S, 44 genes were upregulated and 173 genes were downregulated at stage T1 in P2M. A total of 209 genes and 261 genes were identified to be respectively upregulated and downregulated at stage T2 in P2M. The largest difference existed between P2M and P2S at stage T3, with 916 upregulated genes and 439 downregulated genes identified in P2M. At stage T4, 326 genes were upregulated and 470 genes were downregulated in P2M (Fig. 2b).

**Figure 3:**
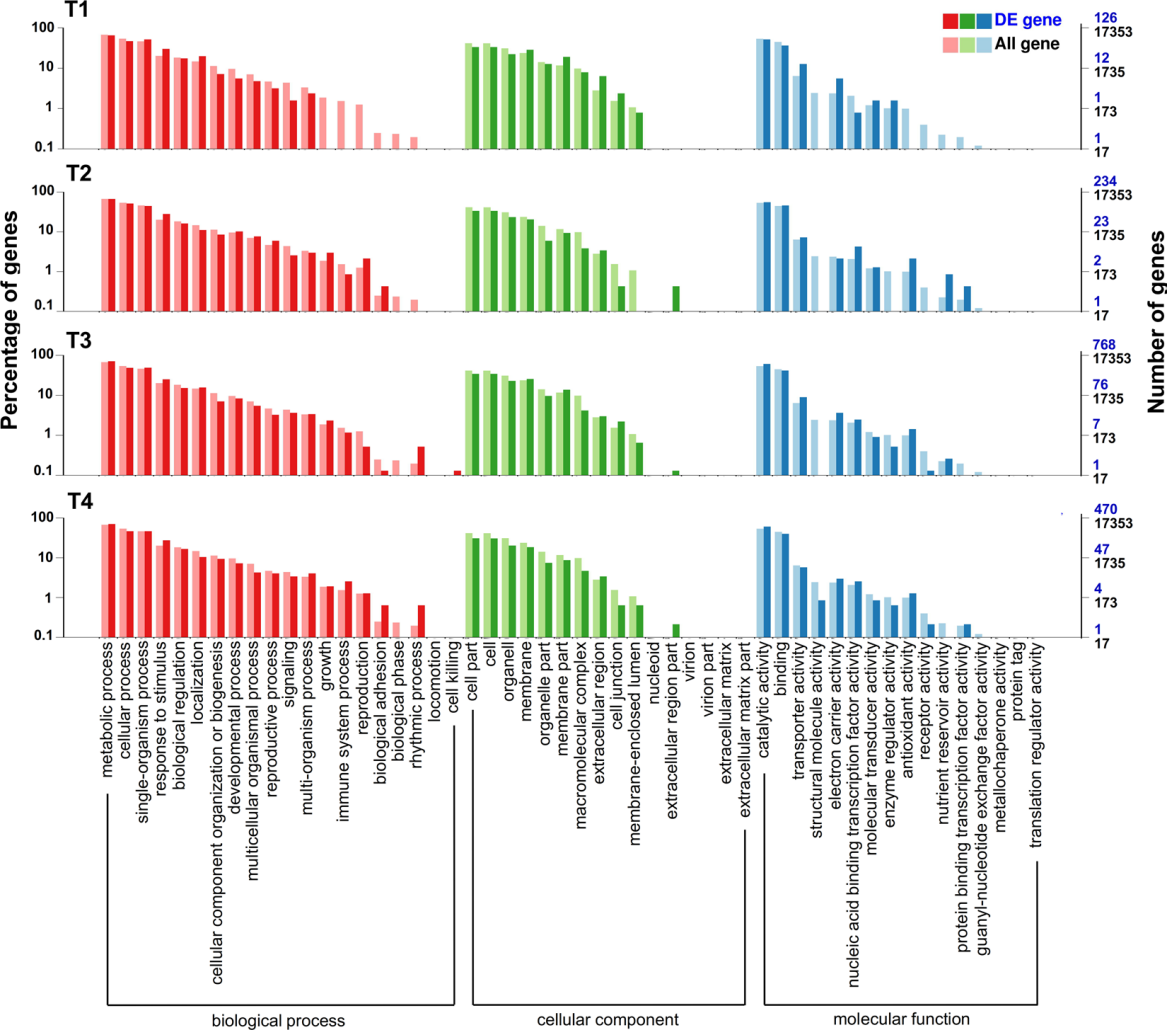
Go functional classification of DEGs between P2M and P2S. The x-axis represents the annotations of GO terms. The left y-axis shows the percentages of the genes and the right y-axis shows the number of the genes. GO terms belongs to biological process, cellular component and molecular function are highlight with red, green and blue color respectively.

To classify the functions of the DEGs, gene ontology (GO) classification and enrichment analysis was performed. A total of 126, 234, 768, and 470 DEGs were categorized into 52 major functional groups at stages T1, T2, T3, and T4, respectively (Fig. 3). The molecular function (MF) of the DEGs mainly embodied catalytic activity, binding, transporter activity, and electron carrier activity. The DEGs categorized as functioning in a biological process (BP) were highly represented in metabolic process, cellular process, single-organism process and response to stimulus. The DEGs that functioned as a cellular component (CC) had some role mainly in cell parts, cells, and organelles at the four stages. However, the differential expression of some genes occurred at different stages (Fig. 3). Within the BP category, the identified DEGs were embodied in growth, immune system process, reproduction, biological adhesion, and rhythmic process at the latter developmental stages. Similarly, DEGs with a CC role were only differentially expressed in the extracellular region during the latter three stages. DEGs with a MF involving structural molecular activity were only detected at stage T4, and regarding receptor activity, at stages T2 and T3. Additionally, when using GO enrichment analysis, it was found that, at the latter three stages, the terms “response to stress” and “response to abiotic stimulus” were significantly enriched in BP, and “UDP-glucosyltransferase activity” was significantly enriched in MF (Table S3). The terms “organic phosphonate transmembrane-transporting ATPase activity” and “mitochondrial proton-transporting ATP synthase complex, catalytic core F(1)” were significantly enriched at stages T2 and T3 (Table S3).

**Figure 4:**
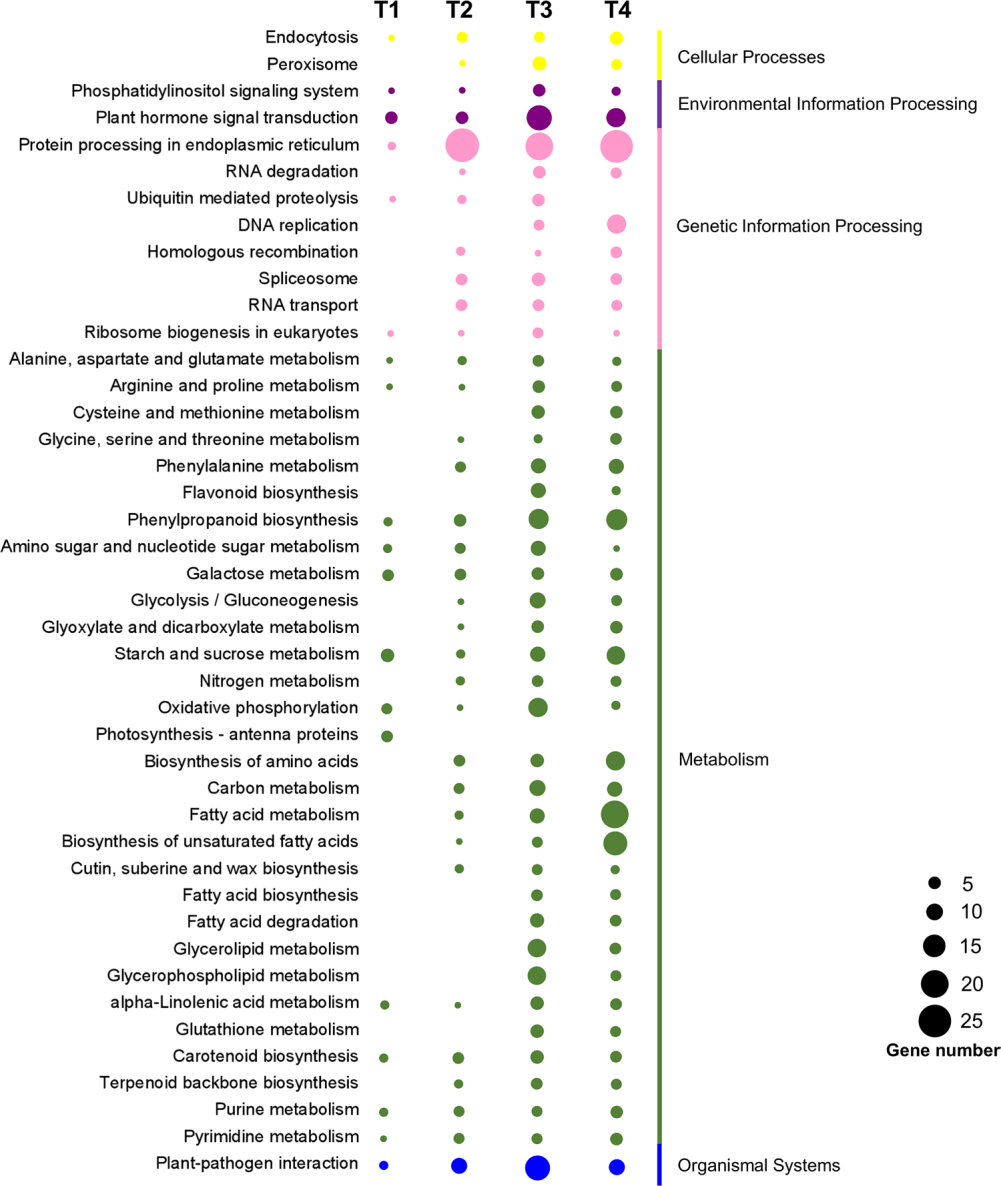
KEGG classification of DEGs between P2M and P2S. The horizontal axis represents four developmental stages. The vertical axis shows the annotations of the KEGG pathways. Different colors represent different KEGG categories. The circle represents the number of genes.

To further explore the biological function of DEGs, we conducted KEGG pathway analysis of all DEGs. Pathways with *P* value < 0.05 were defined as significantly enriched pathways of DEGs. A total of 47, 91, 249, and 188 DEGs were respectively assigned to 35, 52, 97, and 83 pathways at stages T1, T2, T3, and T4, respectively (Fig. 4, Table S4). The pathways with the largest number of DEGs were related to protein processing in the endoplasmic reticulum (ER), followed by pathways related to plant hormone signal transduction and then phenylpropanoid biosynthesis. KEGG enrichment analysis revealed that protein processing in the endoplasmic reticulum pathway was enriched at stages T2 and T4. Moreover, the biosynthesis of unsaturated fatty acids and fatty acid metabolism were also enriched at stage T4. A total of 28 and 31 DEGs were involved in plant hormone signal transduction and phenylpropanoid biosynthesis pathways, respectively. Furthermore, 16 genes encoding pentatricopeptide repeat protein were detected to be differentially expressed between P2S and P2M, and seven of them were targeted to mitochondria (Fig. S1).

### DEGs involved in oxidative phosphorylation

**Figure 5:**
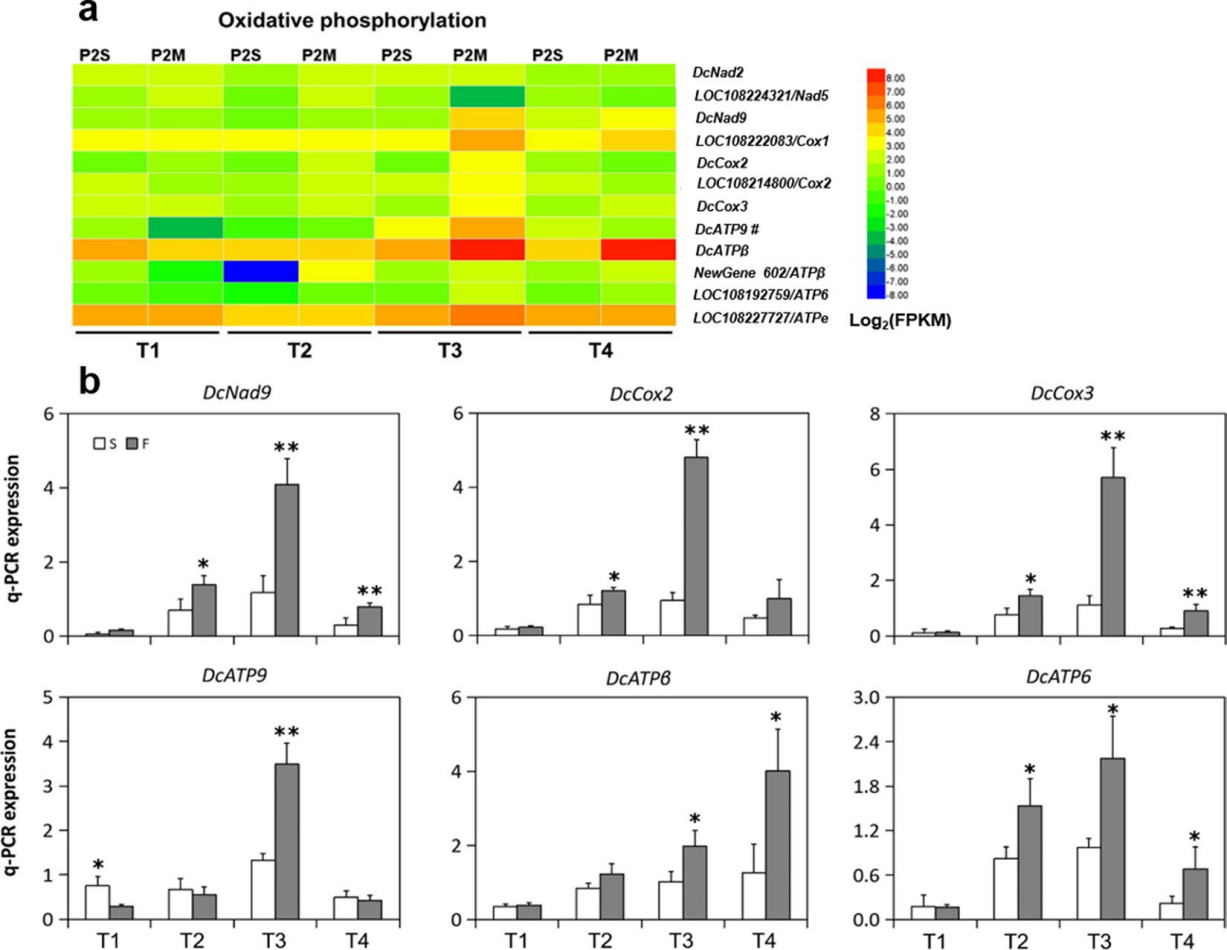
Expression patterns of DEGs related to oxidative phosphorylation and qPCR verification. (**a**) Heatmap of the DEGs. #: The FPKM of *DcATP9* is divided by 1000. (**b**) qPCR analysis of expression profiles of six DEGs. Asterisks indicate that the gene expressions were significantly different between P2S and P2M (^*^P < 0.05; ^**^P < 0.01).

As the center for ATP synthesis, mitochondria play a crucial role in CMS in many plant species^2^. In the present study, 12 genes involved in oxidative phosphorylation were identified to be differentially expressed between P2S and P2M. Among them, two genes encoding NADH dehydrogenase (*DcNad2* and *DcNad5*) were significantly downregulated at stage T2 in P2S when compared to P2M (Fig. 5a, Table S5). Another NADH dehydrogenase gene (*DcNad9*) was distinctly downregulated in P2S at the three latter stages, and had the highest expression at stage T3 in P2M. The qPCR results confirmed that the expression of *DcNad9* in P2M were upregulated 3.5-fold and 2.6-fold at stages T3 and T4, respectively (Fig. 5a,b).

Four transcript genes encoding cytochrome *c* oxidase, *DcCox2*, *DcCox3*, LOC108214800/*Cox2*, and LOC108222083/*Cox1*, were also detected. Notably, it was found that the nuclear gene LOC108214800 was 99% identical with *DcCox2*, which was located in mitochondria (Table S5). The two Cox2-encoding genes and *DcCox3* exhibited similar expression patterns between P2S and P2M. The expressions of *DcCox2* and *DcCox3* in P2M were 5.0-fold and 5.1-fold higher than those in P2S, and reached peak expression at stage T3 in P2M, as verified by qPCR analysis (Fig. 5a,b). Another cytochrome *c* oxidase gene, LOC108222083/*Cox1*, was downregulated only at stage T3 in P2S compared with P2M.

Moreover, five ATPase genes, *DcATP9*, *DcATPβ*, LOC108192759/*DcATP6*, LOC108227727/*ATPe*, and *NoveGene_602* (the homolog of *ATPβ*), also showed similarly downregulated expression patterns at stage T3 in P2S compared to P2M, but showed little difference at stages T1 and T2 (Fig. 5a). Validation of *DcATP6*, *DcATP9*, and *DcATPβ* by qPCR further confirmed our analysis. The expressions of *DcATP6*, *DcATP9*, and *DcATPβ* in P2M were respectively upregulated 2.2-fold, 2.6-fold, and 1.9-fold at stage T3 (Fig. 5b). At stage T1, *DcATP9* was more highly expressed in P2S than in P2M, as determined by RNA-seq and qPCR analysis. These results indicate that the synthesis and transportation of ATP might be disturbed in the CMS line.

### DEGs involved in protein processing in endoplasmic reticulum

**Figure 6:**
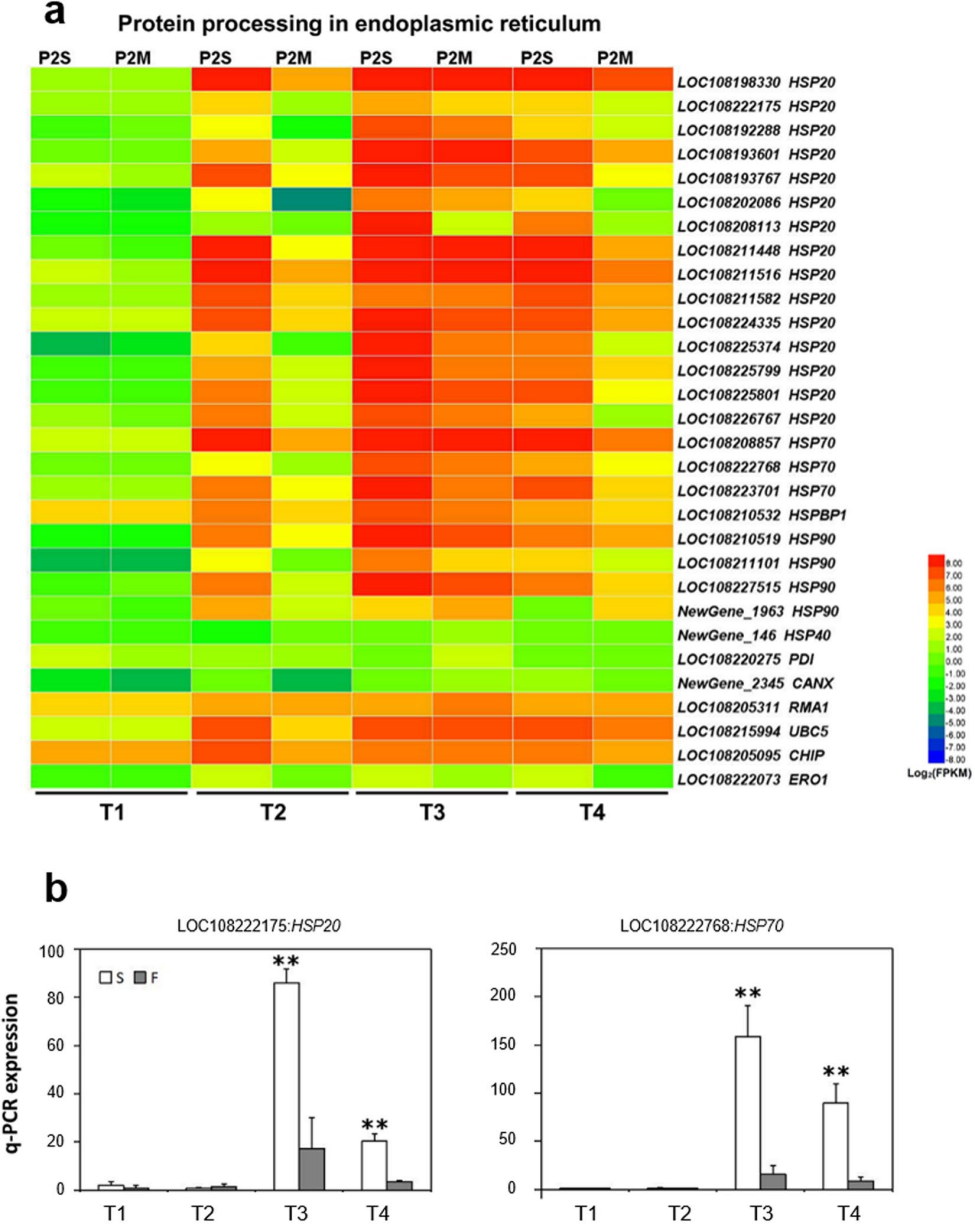
Expression pattern of DEGs assigned to protein processing in endoplasmic reticulum and qPCR analysis. (**a**) Heatmap of the DEGs. (**b**) Expression profile of two DEGs.

Protein processing in the endoplasmic reticulum is one of the most significantly enriched pathways at stages T2 and T4 between P2S and P2M. In this study, 38 DEGs assigned to this pathway were identified as heat shock protein (HSP) genes that were always associated with stress signal pathways, hormone receptors, kinase activity regulation, and intracellular transport^46–48^. Distinct from the expression patterns of DEGs involved in oxidative phosphorylation, most of the HSP genes displayed upregulated expression patterns at the latter three stages in P2S compared with P2M (Fig. 6a).

A total of 15 *HSP20* genes displayed constantly high expression levels from stages T2 to T4 in P2S. By contrast, the expression of these genes in P2M was initially at low levels in stage T2, greatly increased up to the peak expression level in T3, and then declined in T4. However, the expression of *HSP20* genes in P2M was significantly lower than that in P2S throughout the latter three stages. Similarly, three *HSP70* genes (LOC108208857, LOC108222768, and LOC108223701), three *HSP90* genes (LOC108210519, LOC108211101, and LOC108227515), and one gene encoding hsp70-interacting protein (LOC108210532) were also distinctly upregulated from stages T2 to T4 in P2S compared to P2M. Moreover, qPCR analysis showed that the expressions of LOC108222175/*HSP20* and LOC108222768/*HSP70* were significantly upregulated at stages T3 and T4 in P2S, which further confirmed the results of our transcriptome analysis (Fig. 6b). The high expression of HSP genes indicates that flower development in the CMS line might be related to the response to some kind of internal stress.

### MADS-box genes related to floral development

The most conspicuous feature of carrot petaloid CMS is that the stamens are replaced by petal-like organs (Fig. 1i,n). This abnormality is involved in flower organ formation in whorls 2 and 3, and even in whorl 1. In the present study, 16 MADS-box genes were identified, including 3 *DcAP1* genes, *DcPI*, 3 *DcDEF-*like genes, *DcAG*, 3 *DcAG-*like genes, 2 *DcAGL9* genes, *DcAGL12*, *DcSEP1*, and *DcSEP1-*like gene (Table 1).

**Table 1:** Mapping position of MADS-box genes.

Gene	Gene ID	Chromosome	Stop	Length	Protein name
*DcAP1-1*	LOC108197700	8	29718739	244	APETALA 1-1
*DcAP1-2*	LOC108200812	9	19984573	294	APETALA 1-2
*DcAP1-3*	LOC108206004	2	32258267	242	APETALA 1-3
*DcPI*	LOC108217347	4	22420513	444	PISTILLATA
*DcDEFL-1*	LOC108211719	3	12946841	223	DEFICIENS-like-1
*DcDEFL-2*	LOC108220542	5	20185214	218	DEFICIENS-like-2
*DcDEFL-3*	LOC108223743	5	15121726	233	DEFICIENS-like-3
*DcAGL-1*	LOC108214703	1	43593516	267	AGAMOUS-like-1
*DcAGL-2*	LOC108197540	8	16886910	253	AGAMOUS-like-2
*DcAGL-3*	LOC108192538	a	211595	244	AGAMOUS-like-3
*DcAG*	LOC108225552	6	24188878	273	AGAMOUS
*DcAGL9-1*	LOC108206937	2	34789812	242	AGAMOUS-like 9-1
*DcAGL9-2*	LOC108197555	8	31597982	253	AGAMOUS-like 9-2
*DcAGL12*	LOC108196138	1	15801192	205	AGAMOUS-like 12
*DcSEP1*	LOC108214218	3	31493775	244	SEPALLATA 1
*DcSEP1L*	LOC108194187	7	27433047	251	SEPALLATA 1-like

**Figure 7:**
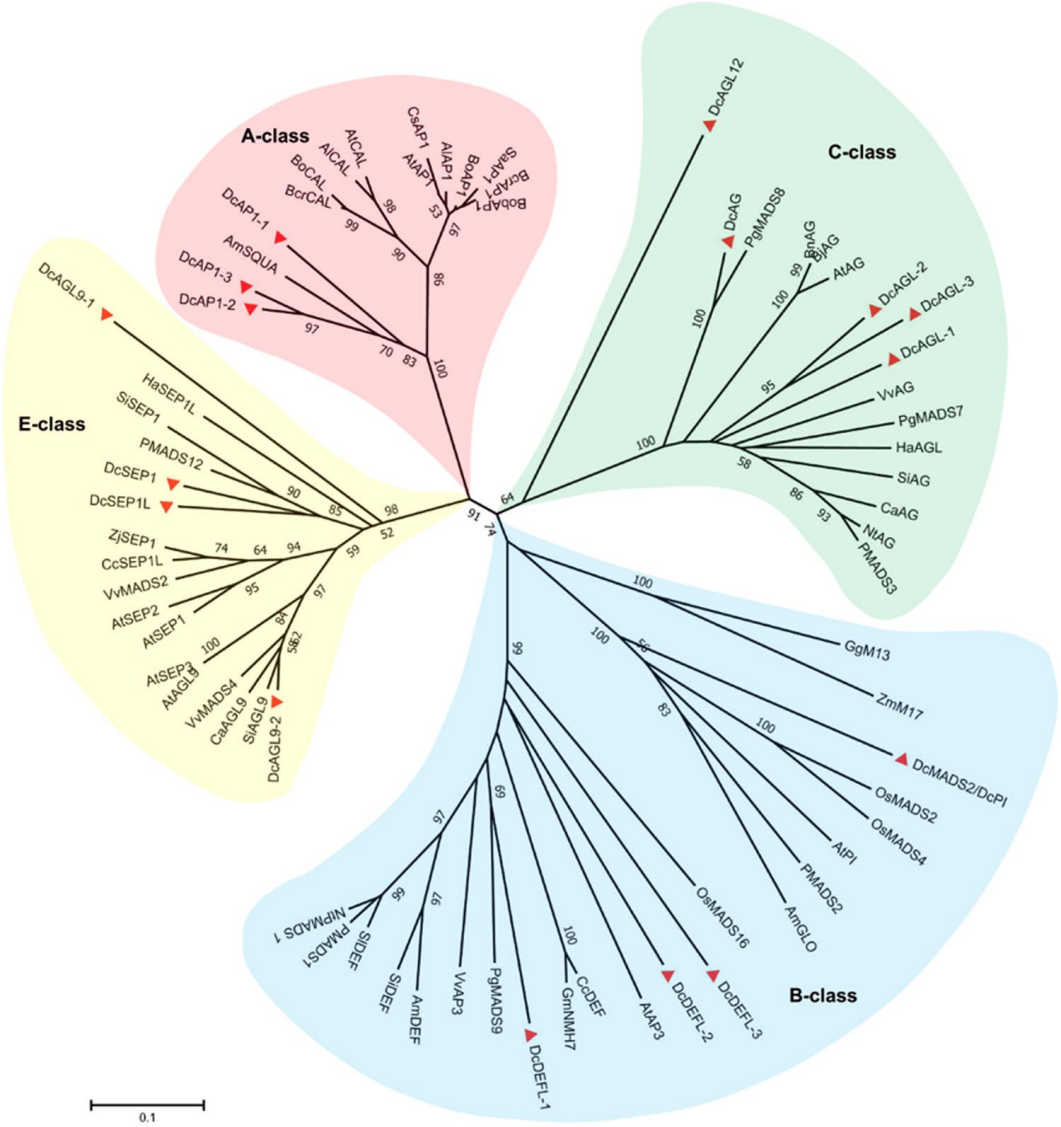
Evolutionary relationships of MADS-box genes. A, B, C and E class MADS-box genes were highlighted with a red, blue, green and yellow circle respectively. Al (*Arabidopsis lyrata*), Am (*Antirrhinum majus*), At (*Arabidopsis thaliana*), Bcr (*Brassica rapa*), Bj (*Brassica juncea*), Bn (*Brassica napus*), Bo (*Brassica oleracea*), Ca (*Capsicum annuum*), Cc (*Cajanus cajan*), Cs (*Citrus sinensis*), Dc (*Daucus carota*), Gg (*Gnetum gnemon*), Gm (*Glycine max*), Ha (*Helianthus annuus*), Nt (*Nicotiana tabacum*), Os (*Oryza sativa*), P (*Petunia hybrida*), Pg (*Panax ginseng*), Sa (*Sinapis alba*), Sl (*Solanum lycopersicum*), Si (*Sesamum indicum*), Vv (*Vitis vinifera*), Zj (*Ziziphus jujuba*), Zm (*Zea mays*).

To analyze the function of these MADS-box genes, they were subjected to unrooted phylogenetic analysis with putative equivalents from 23 plant species based on 70 protein sequences (Fig. 7). The results suggest that *DcMASD2/DcPI* was grouped into the B class, subclustered with *AtPI* and *AmGLO*, and separated from *AtAP3* and *AmDEF*. Three *DcAP1* genes with 71.9% identities were assigned to the A class, adjacent to *AmSQUA*. Three *DcDEF*-like genes belonged to another subtree of the B class and were grouped with *AtAP3* and *AmDEF*. *DcAG*, three *DcAG-*like genes, and *DcAGL12* were assigned to the C class, clustered with *AtAG*, and were contiguous with *VvAG*, an *AG* gene in grapevine (*Vitis vinifera*). The identities of three *DcAG*-like genes were 78.6%. *DcSEP1*, *DcSEP1L*, and two *DcAGL9* were grouped into the E class. No D-class genes were identified in carrot.

**Figure 8:**
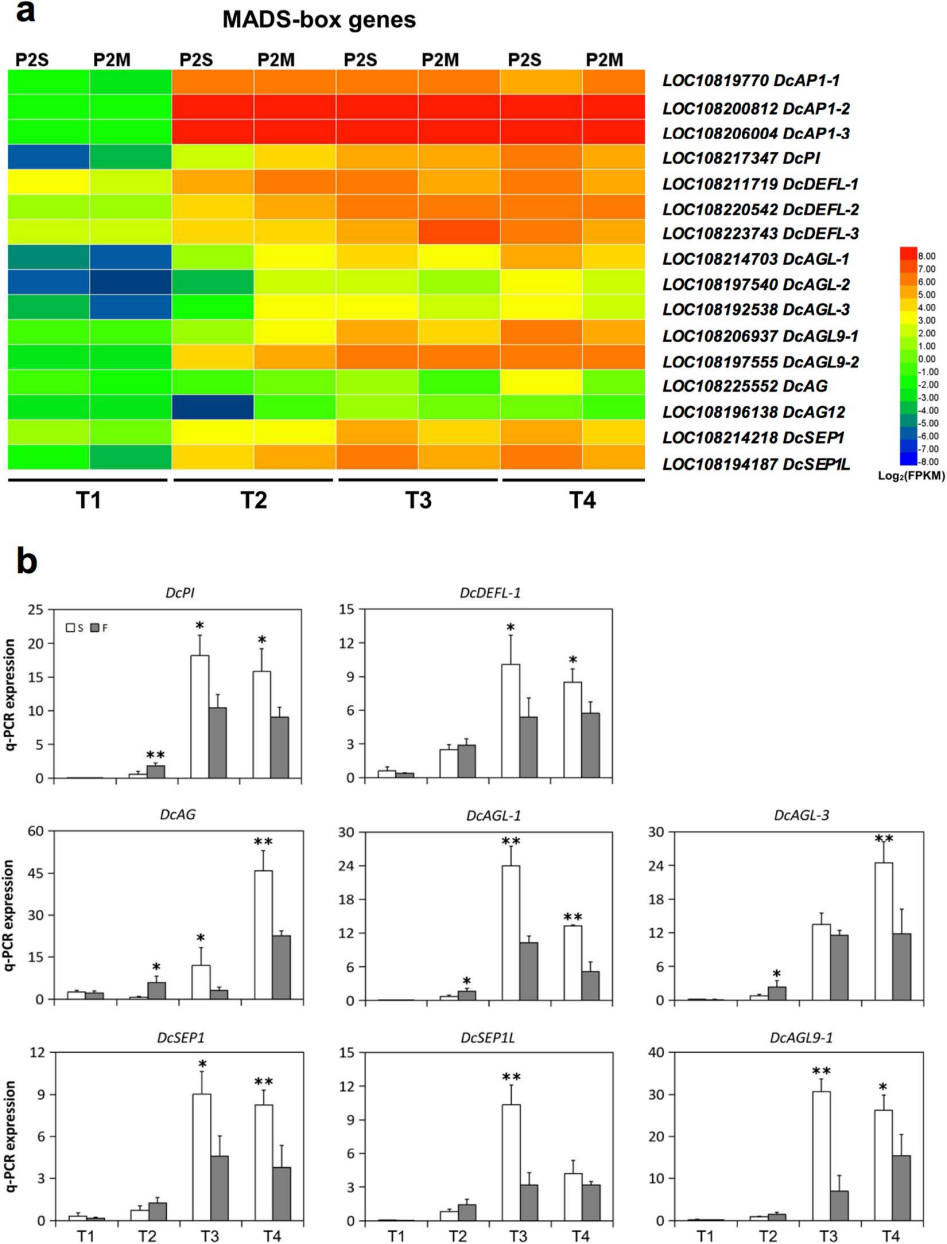
Expression profiles of DEGs related to regulation of flower development and qPCR analysis. (**a**) Heatmap of the DEGs. (**b**) qPCR analysis of expression patterns of eight MADS-box genes.

On the basis of the above analysis, we further analyzed the expression patterns of these MADS-box genes. Nine of them were detected to be differentially expressed between P2S and P2M (Fig. 8a). Little significant difference in expression was found for any of these MADS-box genes between P2S and P2M at stage T1, and the majority of them exhibited extremely low expression levels. It was also found that most of the MADS-box genes were significantly downregulated at stage T2 in P2S as compared to P2M, including *DcPI*, three *DcAG-*like genes, *DcAGL9-1*, and *DcAGL12*. However, conversely, the expression levels of all MADS-box genes except for *DcAP1-1*, *DcAP1-2*, and *DcDEFL-3* increased at stage T3 in P2S, and exceeded the expression level of the same genes in P2M; however, these upregulated expression patterns were not considered statistically significant. Similarly, four MADS-box genes were significantly upregulated at stage T4 in P2S compared with P2M, including *DcDEFL-3*, *DcAGL-1*, *DcAGL-3*, and *DcAG*. To validate the expression pattern of DEGs by RNA-seq, eight MADS-box genes (*DcPI*, *DcDEFL-1*, *DcAG*, *DcAGL-1*, *DcAGL-3*, *DcAGL9-1*, *DcSEP1*, and *DcSEP1L*) were selected for qPCR analysis (Fig. 8b). The results showed that all of the selected MADS-box genes were expressed at a low level at stage T1 in both P2S and P2M, and showed no significant difference. However, the expression of the B-class gene *DcPI* in P2S was downregulated 3.2-fold at stage T2 compared to P2M. *DcAG*, *DcAGL-1*, and *DcAGL-3* exhibited downregulated expression patterns at stage T2 in P2S, which coincided with the expression of *DcPI*. On the contrary, the expression levels of all selected MADS-box genes in P2S greatly increased at stage T3, and were significantly higher than the expression levels in P2M. The expressions of *DcSEP1*, *DcSEP1L*, and *DcAGL9-1* were downregulated at stage T2, however, this change was not significant, though they were significantly upregulated at stage T3 in P2S. The qPCR results not only verified the RNA-seq results but also showed that the majority of these DEGs displayed similar expression tendencies at the same stages.

## Discussion

Petaloid CMS is widely used in carrot breeding, and greatly promotes the development of carrot hybrid varieties^13^. To date, several studies have been performed on the characterization of petaloid CMS using different methods^15–17,19^. However, these studies were mainly focused on the differences in some mitochondrial genes between carrot CMS and fertile materials, and few studies have been carried out at the whole-transcriptome level. In this study, using the high-quality carrot genome^45^, we conducted comparative transcriptomic analysis between the petaloid CMS line and its maintainer line at four flower developmental stages in order to investigate the regulatory network related to CMS and flower organ development. To understand the process of flower formation and to perform a comprehensive transcriptional analysis during flower development, we firstly observed and analyzed the differentiation of flower organs in lines P2S and P2M throughout four developmental periods (Fig. 1). Some considerable differences were identified between the four flower developmental stages, however, little difference was observed between the two lines which is conscious with the previous observation that petaloid florets changed till organ primordia in whorl 3 emerged^40^, indicating that variation in the expression of genes crucial to CMS and flower development can be detected before the morphological change of floral organs in P2S and P2M.

In total, 426.86 million pair-end reads were generated, and the expression of more than 20,000 genes could be detected in each sample of P2S and P2M (Table S6). This large-scale transcriptome analysis provided us with a valuable resource not only for analyzing the regulatory genes related to petaloid CMS, but also for illuminating the developmental process of flower organ formation on a global level. The total number of DEGs gradually increased in parallel with the change of developmental stage, indicating that the regulation network of the male reproductive development process became more sophisticated. Moreover, the number of upregulated genes at stage T3 increased suddenly, and outnumbered the downregulated genes by more than two-fold compared to other stages (Fig. 2b), since floret primordia germinated at this stage and more genes were involved in metabolic processes and cellular processes at this developmental stage (Figs 3 and 4). GO functional analysis revealed that organic phosphonate transmembrane-transporting ATPase activity and mitochondrial proton-transporting ATP synthase complex were enriched, which suggested that alternation of the expression of ATPase-related genes might have an influence on energy metabolism between P2S and P2M. Additionally, KEGG analysis showed that many genes were involved in oxidative phosphorylation, protein processing in the endoplasmic reticulum, and others (Fig. 4), which suggests that these pathways might play important roles in carrot petaloid CMS and flower development. Furthermore, the expression pattern of DEGs and qPCR analysis indicated that most genes involved in these pathways, such as *DcNad9*, *DcCox2*, *DcAG*, and *DcAGL-1*, start to show different expression patterns at stages T2 and T3 (Figs 5–7), indicating that stage T2 might be a key stage during the regulation of petaloid CMS.

Oxidative phosphorylation, also known as the mitochondrial electron transport chain, is one of the important energy production processes in mitochondria, and is crucial for normal plant development, especially flower organ development^49,50^. NADH dehydrogenase, cytochrome *c* oxidase, and ATPase are key elements of enzyme complexes in the mitochondrial respiratory chain. The activities of these genes are correlated with ATP production. Energy deficiency caused by components of oxidative phosphorylation is one of the important factors responsible for CMS and male flower organ development^7,8,51,52^. In this study, three genes encoding NADH dehydrogenase, four genes encoding cytochrome *c* oxidase, and four genes encoding ATPase were detected and analyzed; *ATP*8 was detected without difference, which is in agreement with the results of Robison and Wolyn^18^ (Fig. 5). During the four developmental stages, the expression of most DEGs in P2M significantly increased at stages T2 and T3, and reached their peak expression at T3. By contrast, the expression of DEGs in P2S exhibited continuously low levels from stages T2 to T4. Thus, our results indicate that ATP production might be greatly inhibited in the CMS line due to the decline in transcript levels of mitochondrial respiratory chain components, and would therefore not be able to meet the requirements for normal stamen development. Interestingly, we also identified two genes, *DcCox2* and LOC108214800, which shared 99% identity and were respectively located in the mitochondria and nucleus. This finding is consistent with the phenomenon that endosymbiotic genes are transferred from organelles to the nucleus^53,54^, which suggests that there might be an endosymbiotic gene process in the carrot genome.

The endoplasmic reticulum is an important subcellular organelle where proteins are folded with the help of lumenal chaperones^55^. Based on KEGG enrichment analysis, 38 DEGs were found to be enriched in protein processing in the endoplasmic reticulum, and most of them were classified as HSPs. HSPs function as molecular chaperones to regulate the folding, localization, and degradation of proteins in the endoplasmic reticulum^56^. More importantly, HSPs play vital roles in signal transduction during stress response^46,47^. Our results also showed that response to stress and abiotic stimulus were enriched in biological processes at stages T2–T4 (Table S3). Abiotic stresses, such as cold and heat, have great effects on floral organ development, especially male flower organs, and HSPs therefore play a vital role in maintaining normal fertility under abiotic stress conditions^57^. Stamen petaloidy in P2S might be considered as an intrinsic stress situation for plants. Thus, we presume that when P2S detects signals of stamen petaloidy and its own response to this internal stress is consequently triggered, resulting in the upregulated expression of HSP genes.

Petaloid CMS in carrot is characterized by male organ abnormality caused by the homeotic conversion of stamens into greenish petals (Fig. 1i,n). Previous studies in *Arabidopsis* suggested that the identity of floral organs during the developmental process was controlled by MADS-box genes^58^. According to the floral quartet model, class A, B, and E genes specify petals and class B, C, and E genes specify stamens^32^. In this study, 16 MADS-box genes were found, 9 of which were found to be differentially expressed between P2S and P2M (Table 1). Interestingly, there are A-, B-, C-, and E-class MADS-box genes in carrot, however, D-class genes are absent (Fig. 7). When the inflorescence primordia started to differentiate at stage T2, we observed a drastic decrease in the transcription levels of the B-class gene *DcPI* and three C-class genes *DcAG*, *DcAGL-1*, and *DcAGL-3* in P2S (Fig. 8a,b). In homeotic *Arabidopsis* and *Antirrhinum* mutants with impaired B-class genes, petals are replaced by sepals and stamens are substituted by carpels^33,34^. The petaloid flowers strongly resemble homeotic *Arabidopsis* and *Antirrhinum* mutants with impaired C-class genes based on their petal-like structures in whorl 3^33,34^. In this study, three C-class *DcAG-*like genes exhibited similar downregulated expression patterns at stage T2 in P2S (Fig. 8a,b). Thus, our results suggest that the downregulated transcript levels of B- and C-class genes might play a key role in the homeotic conversion of stamens into petal-like organs in carrot. However, the expressions of B-, C-, and E-class genes in P2S sharply increased at stages T3 and T4 as compared to stage T2, and were significantly higher than those in P2M. The simultaneously upregulated expression patterns of *DcPI*, *DcDEF*, *DcAG*, *DcAGLs*, and *DcSEP1s* at stages T3 and T4 in P2S indicates their interdependence as described in four-quarter model in *Arabidopsis*, and that B-factors interacted with C- and E-factors to regulate stamen and petal development^32^. Additionally, the highly increased expression patterns of these MADS-box genes with high expression in P2S are in agreement with the upregulated expression profiles of HSP genes at stages T3 and T4 in P2S. We hypothesized that the low transcript abundance of B- and C-class genes in P2S at stage T2 might be associated with the petaloidy of stamens, and that T2 might be a crucial stage for stamen development in carrot. When plants receive stamen petaloidy signals at stage T2, they might regard these signals as internal stress, and thus trigger the upregulation of HSP genes and MADS-box genes to resist the development of male organ abnormality. This could explain the observation that there was a small proportion of revertant plants with normal stamens (3.4%) in P2S.

In general, previous studies have reported that mitochondrial genes could affect the expression of nuclear genes that are related to floral organ development^23,26,59^. In this study, our results indicate that several key genes involved in oxidative phosphorylation, such as NADH dehydrogenase, cytochrome *c* oxidase, and ATPase, show continuously low expression from stage T2, which caused energy deficiency in P2S (Fig. 5), and that the expression of *DcPI* and *DcAG-*like genes greatly decreased at stage T2 in P2S (Fig. 8), which might be one factor causing stamens to convert into petals. However, it is confusing that the MADS-box genes were expressed to a significantly higher degree in P2S than P2M at stages T3 and T4, while the expression of genes involved in oxidative phosphorylation was still low in P2S. Stamen petaloidy might be interpreted as an intrinsic stress by plants, and contribute to the upregulated expression of HSP genes and MADS-box genes at stages T3 and T4 in P2S (Figs 6 and 8), resulting in the occurrence of some fertile revertants. The results of this study provide an improved foundation for further research into the molecular mechanisms of carrot petaloid CMS and flower development. Future work focused on characterizing the function of these candidate genes will be helpful for understanding CMS and flower development in carrot.

## Methods

### Plant materials

The petaloid CMS line (P2S) at BC_1_S_4_M_6_ and its maintainer line (P2M) at F_2_S_4_M_6_ were used in this study, these two lines have very similar genetic background except the different male flower organs. Seeds corresponding to these two accessions were directly sown in the field at the Langfang station of the Chinese Academy of Agricultural Sciences on 28 July 2016. Roots were harvested in late November, stored under cold store, and transplanted in the field in late March 2017. Considering that gene expression usually displays significant differences prior to morphological changes, we chose the period when the two accessions had gone through vernalization as the first sampling stage (T1)^14^, in order to ensure a more comprehensive detection of gene expression. For the next three sampling stages, T2, T3, and T4, we chose periods according to the previous phenotype observation of flowers^40^ when the transverse length of umbels had respectively reached 2.0, 3.0, and 4.0 mm. The stem apex samples of lines P2S and P2M at stage T1, and umbels grown at lateral branches at the latter three stages, were collected for RNA-seq and qPCR analysis using three biological replicates. All samples were frozen in liquid nitrogen and stored at −80 °C until needed.

### Scanning electron microscopy of stem apex and inflorescence

Samples of P2S and P2M at different developmental stages were dissected under an anatomical lens, and then transferred to 2.5% glutaraldehyde fixative and fixed for 4 h at room temperature with intermittent shaking. Then, the samples were washed three times in 0.1 M phosphate buffer (pH 7.2) before the addition of aqueous osmium tetroxide and post-fixed in 1% aqueous osmium tetroxide for 2 h. After that, materials were washed three times with ddH_2_O to remove the remaining osmium tetroxide and were then dehydrated through 20 min exposures to a series of increasing ethanol gradients; the last dehydration with 100% ethanol needed to be repeated three times to ensure that samples were thoroughly dehydrated. After dehydration, samples were submerged in isoamyl acetate for 30 min to replace the ethanol. Subsequently, samples were dried using the CO_2_ critical point drying method. Then, samples were mounted on a scanning electron microscopy (SEM) sample platform and coated with palladium–gold using a Hitachi IB-5 ion plating instrument (Hitachi, Tokyo, Japan). The prepared samples were viewed and photographed using a Hitachi SU8000 SEM (Hitachi, Tokyo, Japan).

### RNA extraction and library construction for transcriptome sequencing

Total RNA from stem apex samples and young umbels was isolated using the RNAprep Pure Plant Kit (TianGen, Beijing, China). The assessments of RNA quantity and quality were followed by Wang *et al*. method^60^. Sequencing libraries were constructed using the NEBNext Ultra^TM^ RNA Library Prep Kit for Illumina (NEB, Ipswich, MA, USA) and performed as described in Ou *et al*.^61^. The library construction and Illumina sequencing were performed on an Illumina HiSeq 2500 platform by Biomarker Technologies Co., Ltd. (Beijing, China).

### Differential expression analysis and functional annotation

After removal of the adaptor sequences and low-quality sequence reads, raw sequences were transformed into clean reads. These clean reads were then mapped to the reference genome of carrot^45^ using TopHat2^62^. Gene expression levels were estimated in fragments per kilobase of transcript per million fragments mapped (FPKM). Prior to differential gene expression analysis, for each sequenced library, the read counts were adjusted using the edgeR program package^63^. The resulting FDR was adjusted using the posterior probability of being differential expression (PPDE). FDR <0.01 and |log2(fold change)|≥1 were set as the threshold for significant differential expression. Venn diagrams of DEGs were created using VENNY (http://bioinfogp.cnb.csic.es/tools/venny/index.html).

Gene function was annotated based on the following databases: the NCBI non-redundant protein sequences (nr) database, the NCBI nucleotide sequences (nt) database, the protein family (Pfam) database, the KOG/COG database, the Swiss-Prot database, the Gene Ontology database, and the KEGG database. GO enrichment analysis of DEGs was implemented by the GOseq R package-based Wallenius non-central hypergeometric distribution^64^. KEGG pathway enrichment analysis was based on all detected DEGs between male sterile and fertile samples using the KEGG pathway database^65^. KOBAS software was used to test the statistical enrichment of differentially expressed genes in KEGG pathways^66^.

### Phylogenetic analysis of mads-box genes

The 70 protein sequences of the MADS-box genes in 24 representative species (Fig. 7) were downloaded from the NCBI database. Multiple sequence alignment was conducted using ClustalW. Evolutionary analyses were performed using MEGA 7.0^67^, using the neighbor-joining method to infer evolutionary history and p-distance method to compute distance. The bootstrap consensus tree inferred from 1000 replicates was taken to represent the evolutionary history of the taxa^68^.

### Quantitative real-time PCR (qPCR) analysis

To verify the expression of DEGs identified by RNA-seq, qPCR analyses were performed on samples from the CMS line and its maintainer line at four developmental stages. The RNA samples used for qPCR were identical to those used for RNA-seq. The qPCR reaction procedure and analysis of relative expression level followed that of Wang *et al*.^60^ using a Bio-Rad CFX96 instrument (Bio-Rad, Hercules, CA, USA). Gene-specific primers (Table S7) for qPCR were designed using Primer Premier 5.0 based on the candidate gene sequences. Three biological replicates and three technical replicates were conducted for each sample. The relative expression level of each gene was calculated using the 2^−ΔΔCt^ method and all data are shown as mean ± standard deviation between three biological replicates^69^.

## Supplementary information

Supplementary figrues

supplementary tables
