# Supplementary material for: Differentially Expressed Genes between Carrot Petaloid Cytoplasmic Male Sterile and Maintainer during Floral Development
Source: Sci Rep. 2019 Nov 22;9:17384. doi: 10.1038/s41598-019-53717-x (PMC6874560; doi:10.1038/s41598-019-53717-x)
Supplement: Supplementary file 1 — Supplementary figrues [file 41598_2019_53717_MOESM1_ESM.docx]

**Differentially Expressed Genes between** **Carrot Petaloid Cytoplasmic Male Sterile and Maintainer during Floral Development**

Bo Liu^1+^, Chenggang Ou^1+^, Shumin Chen^1+^, Qiongwen Cao^1^, Zhiwei Zhao^1^, Zengjian Miao^2^, Xiaoping Kong^2^ and Feiyun Zhuang^1^*

^+^ These authors contributed equally to this work and share the first authorship.

^1^ Key Laboratory of Horticultural Crop Biology and Germplasm Innovation, Ministry of Agriculture; Institute of Vegetables and Flowers, Chinese Academy of Agricultural Science

No. 12 Nanda Street, Zhongguan Cun, Haidian District, Beijing 100081, China

^2^ Xining Institute of Vegetables, Xining

No. 4 Weisan Road, Biological Industry Park, Xining 810016, Qinghai

*Corresponding author. E-mail address: [zhuangfeiyun@caas.cn](mailto:zhuangfeiyun@caas.cn)

**Supplementary Figure**


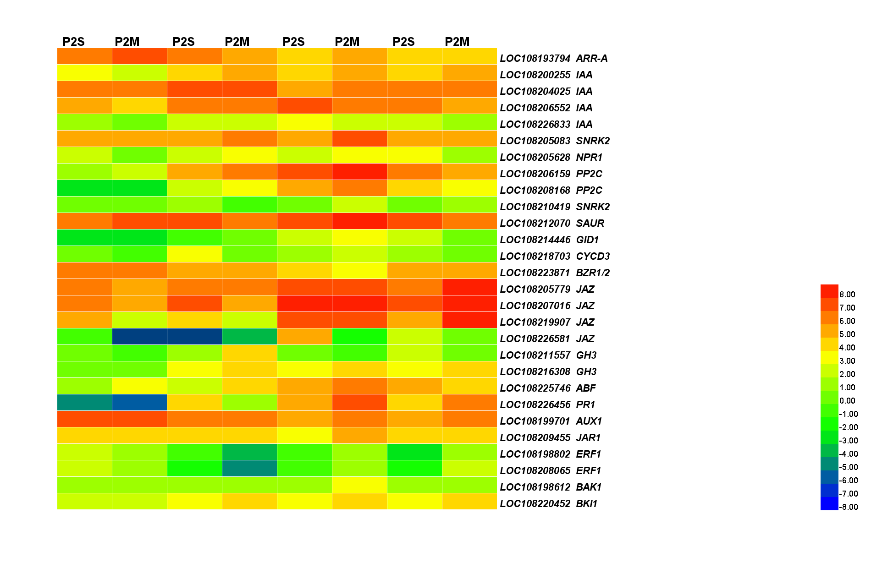


**Plant hormone signal transduction**

**T2**

**T3**

**T4**

**T1**

**Phenylpropanoid biosynthesis**


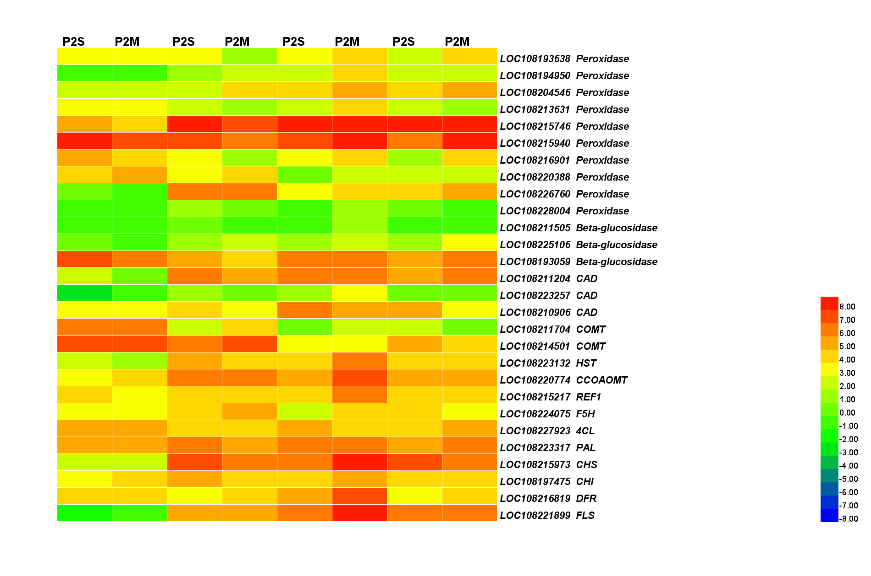


**T2**

**T3**

**T4**

**T1**

**Pentatricopeptide repeat Protein**


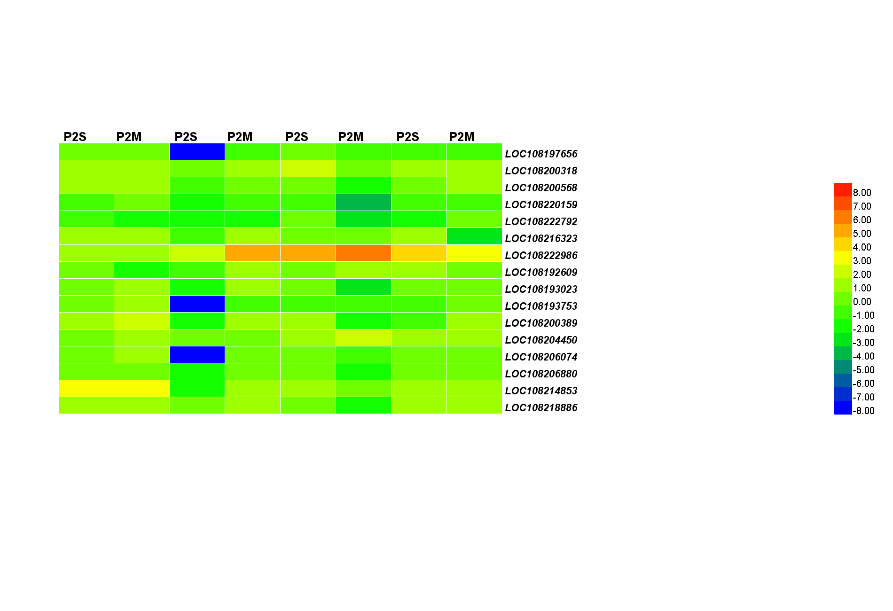


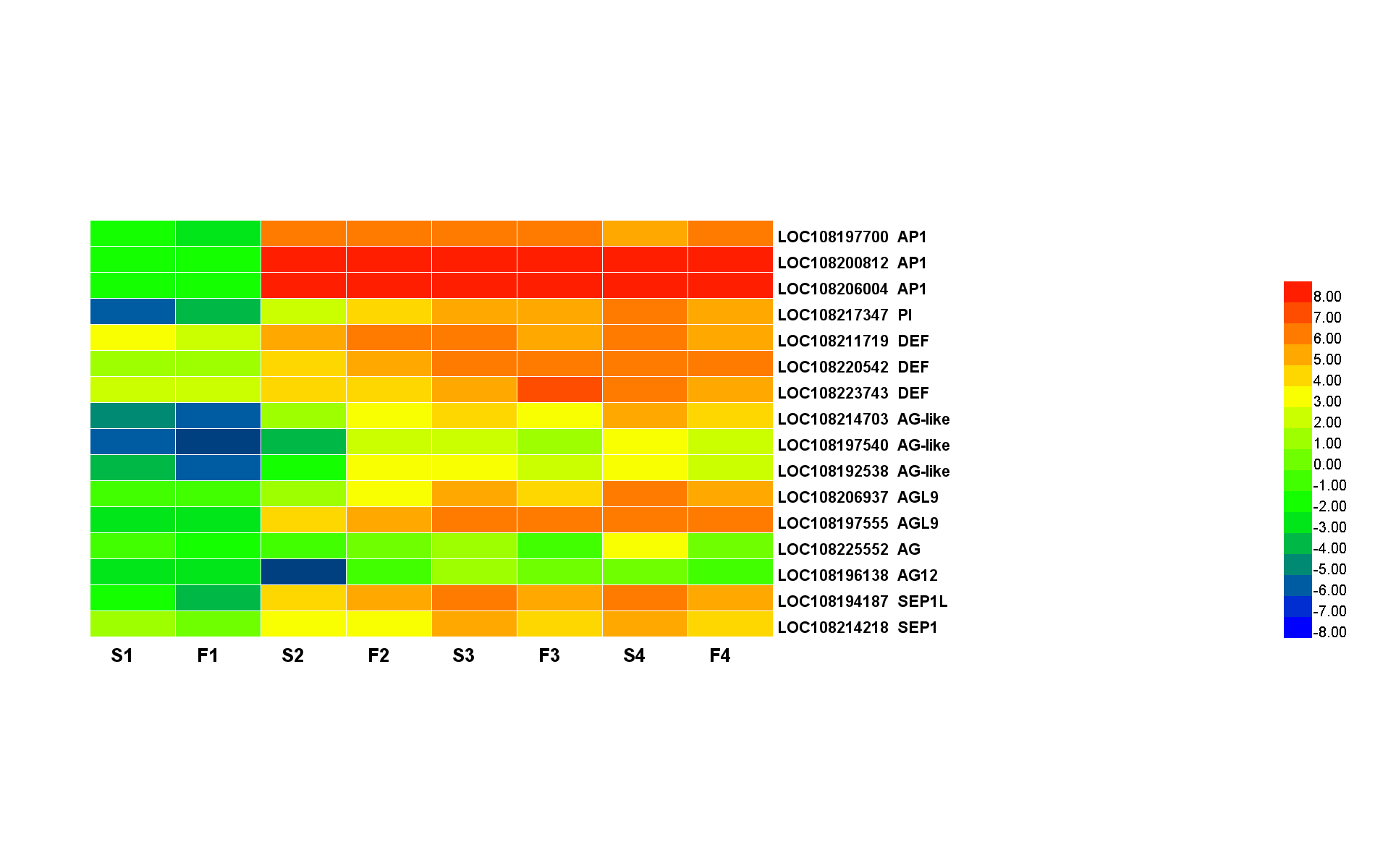


**Log_2_(FPKM)**

**T2**

**T3**

**T4**

**T1**

**Figure S1.** Heatmap of DEGs involved in plant hormone signal transduction, phenylpropanoid biosynthesis and pentatricopeptide repeat protein.
